# Quantifying network properties in multi-electrode recordings: spatiotemporal characterization and inter-trial variation of evoked gamma oscillations in mouse somatosensory cortex *in vitro*

**DOI:** 10.3389/fncom.2013.00134

**Published:** 2013-10-16

**Authors:** Cristian Carmeli, Paolo Bonifazi, Hugh P. C. Robinson, Michael Small

**Affiliations:** ^1^Laboratoire de Recherche en Neuroimagerie, Department of Clinical Neurosciences, Centre Hospitalier Universitaire Vaudois and University of LausanneLausanne, Switzerland; ^2^Department of Physiology, Development and Neuroscience, University of CambridgeCambridge, UK; ^3^School of Physics and Astronomy, Tel-Aviv UniversityTel-Aviv, Israel; ^4^School of Mathematics and Statistics, The University of Western AustraliaCrawley, WA, Australia

**Keywords:** gamma oscillations, acute cortical slices, multi-electrode arrays, local field potentials, spatial synchronization, functional connectivity

## Abstract

Linking the structural connectivity of brain circuits to their cooperative dynamics and emergent functions is a central aim of neuroscience research. Graph theory has recently been applied to study the structure-function relationship of networks, where dynamical similarity of different nodes has been turned into a “static” functional connection. However, the capability of the brain to adapt, learn and process external stimuli requires a constant dynamical functional rewiring between circuitries and cell assemblies. Hence, we must capture the changes of network functional connectivity over time. Multi-electrode array data present a unique challenge within this framework. We study the dynamics of gamma oscillations in acute slices of the somatosensory cortex from juvenile mice recorded by planar multi-electrode arrays. Bursts of gamma oscillatory activity lasting a few hundred milliseconds could be initiated only by brief trains of electrical stimulations applied at the deepest cortical layers and simultaneously delivered at multiple locations. Local field potentials were used to study the spatio-temporal properties and the instantaneous synchronization profile of the gamma oscillatory activity, combined with current source density (CSD) analysis. Pair-wise differences in the oscillation phase were used to determine the presence of instantaneous synchronization between the different sites of the circuitry during the oscillatory period. Despite variation in the duration of the oscillatory response over successive trials, they showed a constant average power, suggesting that the rate of expenditure of energy during the gamma bursts is consistent across repeated stimulations. Within each gamma burst, the functional connectivity map reflected the columnar organization of the neocortex. Over successive trials, an apparently random rearrangement of the functional connectivity was observed, with a more stable columnar than horizontal organization. This work reveals new features of evoked gamma oscillations in developing cortex.

## Introduction

In the last decade, the connectome approach is providing more and more information on the wiring of the nervous system at different spatial scales of investigation, while multi-channel electrical and optical recordings provide a massive amount of data on brain activity dynamics recorded from multiple sites (Friston, 2005; Lebedev and Nicolelis, 2006; Homma et al., 2009; Sporns, 2011). Therefore, there is a need for new theoretical and experimental tools to bridge these different levels (scale) of investigations (Stevenson et al., 2008; Rubinov and Sporns, 2010; Feldt et al., 2011; Bastos et al., 2012). The aim of this paper is to provide a range of techniques that may be applied to elucidate functional relationships among brain regions from multi-electrode recordings of structured cortical tissue. In particular, by studying the dynamics of gamma oscillatory activity, a main brain rhythm (Buzsáki, 2006; Fries, 2009), we concentrate on establishing a clear and general set of tools that minimizes assumptions about network structure.

Oscillatory synchronous activity is a general feature of firing of neuronal networks both in adult animals and during development (Buzsáki, 2006; Uhlhaas et al., 2010). Oscillations are seen *in-vivo* in local field potential and/or EEG signals, in peripheral structures (olfactory bulb), in sensory and motor cortices, in the hippocampus and in the thalamus (Buzsáki and Draguhn, 2004; Buzsáki, 2006). *In vitro*, oscillations have been observed in spontaneous activity or in response to drug application and/or electrical stimulation (Buhl et al., 1998; Traub et al., 2004; Khazipov and Luhmann, 2006). Intracellular recording of single neurons during network oscillations reveals subthreshold membrane potentials fluctuations and action potential timing which is correlated to the oscillation in extracellular field potential (Hasenstaub et al., 2005). Oscillations occur over a very wide range of frequencies (*f*) ranging from 0.05 to 500 Hz, with EEG signals showing a *1/f* power relationship (Buzsáki and Draguhn, 2004). This suggests that perturbations occurring at slow frequencies can cause a cascade of energy dissipation at higher frequencies, and that widespread slow oscillations could modulate faster local events. Within this wide range of frequencies, gamma rhythms (30–80 Hz) have been proposed to play a fundamental role in sensory encoding and processing (Fries, 2009), for example in the olfactory system (Laurent and Davidowitz, 1994), in perceptual binding (Singer and Gray, 1995), motor programming (Murthy and Fetz, 1996), and associative learning (Miltner et al., 1999). In the somatosensory cortex, detection of input frequencies in the gamma range is functionally important (Romo and Salinas, 2003).

It appears that the gap-junctional network of coupled fast-spiking inhibitory interneurons, which forms an electrical syncytium through the layers of the cortex (Galarreta and Hestrin, 1999; Gibson et al., 1999), is a crucial component in the dynamics of gamma oscillations (Fries, 2005; Hasenstaub et al., 2005; Morita et al., 2008). The ability to evoke gamma oscillations in acutely-isolated slices (Traub et al., 2004) makes it clear that local mechanisms in the cortex can suffice to drive gamma oscillations. Interestingly, gamma oscillations have been recorded also in dissociated neuronal cultures which lack the structural organization of intact cortical circuits (Shein Idelson et al., 2010).

To assess how gamma oscillations can structure the timing of network activity and set the context for temporal coding in cortical neurons, it is necessary to understand quantitatively how gamma oscillations form and disperse in space and time and their variability in different activation. In this work, we have electrically evoked and recorded gamma oscillations from the local field potential at multiple sites within acutely-isolated slices of mouse somatosensory cortex, using planar multielectrode arrays (MEAs) (Maeda et al., 1995; Jimbo et al., 1998). We used Hilbert transform and wavelet analysis to resolve the temporal evolution of oscillation frequency and phase at multiple sites, and methods of non-linear time series analysis (Kantz and Schreiber, 1997) to describe the evolution of dynamical synchrony at different sites at the pair-wise level. This allowed us to quantify how synchrony between different regions of the cortical network builds up and decays during an oscillatory event and how synchrony varies when applying repeated stimuli.

The experimental data shows significant trial-to-trial variability in both the raw time series and the evoked gamma oscillations. Nonetheless, the data analysis described above allows us to study the mechanism underlying onset and maintenance of synchronization in different regions within a gamma burst. We find that functional connectivity between multi-electrode locations has a rather non-random structure (more vertically oriented than horizontal), but that the precise configuration of the connections appears random, changing from trial to trial. Hence, we do not explicitly build a network, nor analyse the network properties. The main effect we observe is evident in the extent and dynamics of synchrony as measured operationally in terms of phase synchronization or functional connectivity-by analyzing more elaborate network based properties we would only serve to obfuscate this information. In this report, we focus on the dynamic aspect of transient coupling using a non-linear measure of functional connectivity (phase synchronization). Here, we use functional connectivity in the formal sense to reflect statistical dependencies among neuronal responses. However, we do not attempt to explain this in terms of directed or effective connectivity—or the underlying microcircuits—but simply highlight the challenge to understand the phenomena we report below.

## Methods

### Slice preparation

Neocortical slices have been prepared as described in (Kim and Robinson, 2011). Briefly, 8–12 days old CD-1 mice (Charles River, Margate, UK) were used for brain slice preparation (killed according to UK Home Office guidelines). Following cervical dislocation, the brain was rapidly immersed in ice-cold Ringer's solution of the following composition (in mM): 125 NaCl, 25 NaHCO_3_, 2.5 KCl, 1.25 NaH_2_PO_4_, 2 CaCl_2_, 1 MgCl_2_, and 25 glucose, oxygenated with 95% O_2_, 5% CO_2_ to maintain a pH of 7.4. Three hundred micrometer sagittal slices were cut from one hemisphere. Brain slices were incubated in Ringer's solution on a cotton mesh at room temperaturefor at least 30 min before recording and perfused with oxygenated slicing solution at 30–33°C during recording.

### Electrical recording and stimulation through multi-electrode arrays

A Multichannel Systems (Reutlingen, Germany) MEA60 multielectrode array recording system was used to record simultaneously from 60-channel planar MEAs. Slices were held on the surface of the array using a “harp” of threads stretched across a gold ring (Sakmann and Stuart, 1995). Five biphasic voltage pulses at 200 Hz of amplitude varying between 0.1 and 1 V and duration of 0.1 ms each, were delivered through subsets of the array electrodes using the blanking circuitry built in to the preamplifiers. The particular subsets of electrodes used to deliver the stimuli were selected in order to produce reliable gamma responses. Having been selected, the same set of electrodes was used for all subsequent stimulations and usually comprised one to three electrodes of the available array. The minimal amplitude capable of evoking a gamma burst was applied to the subset of electrodes used for stimulation. Stimulations were delivered with at least 30 s interval in between consecutive stimulations. Removal of the stimulation artifact was performed according to Ruaro et al. (2005).

When synaptic agonists/antagonists were used, drugs were perfused for at least 20 min before applying the stimulation protocol.

The following synaptic agonists/antagonists were used: 2-amino-5-phosphonovalerate (APV; Sigma-Aldrich), Carbachol (Sigma-Aldrich), Carbenoxolone (Sigma-Aldrich), Mefloquine (Sigma-Aldrich), Kainic Acid (Sigma-Aldrich), CNQX (Sigma-Aldrich) and Gabazine (Sigma-Aldrich). In Table 1, we report the receptors and gap junction substrates upon which the drugs act.

**Table 1:** **List of drugs used for testing the synaptic pathways underlying the occurrence and the dynamics of gamma oscillations**.

**Drug**	**Concentration (μM)**	**Receptor agonist**	**Receptor antagonist**	**Blocked gap junction**
Carbachol	50	acetylcholine		
Kainic Acid	10	kainate		
CNQX	10		AMPA kainate	
APV	50		NMDA	
Carbenoxolone	100–200			Unspecific
Gabazine	10		GABA-A	
Mefloquine	25			Mediated by connexin 36–50

The functional role of each drug (agonist, antagonist, blocker) and its targets (receptors, connexins) are specified.

### Current source density

Local field potentials are thought to predominantly stem from dendritic processing of synaptic inputs, but a direct interpretation in terms of the underlying neural activity is difficult (Freeman and Nicholson, 1975). A standard procedure has been to record the field potential at equidistant, linearly positioned electrode contacts vertically penetrating the cortical layers. Under various assumptions, the current source density (CSD) can be estimated from a double spatial derivative of the recorded potentials. Practically, the CSD is estimated according to

$$\sigma \frac{\partial^{2}V}{\partial z^{2}}=-\text{CSD}(z)$$

Here, σ is the homogenous and isotropic conductivity, *z* is the axis defined to be perpendicular to the cortical layers, *V* is the recorded potential, which is assumed to be constant in the in-layer plane. This corresponds to assuming that the neuronal current sources effectively are infinitely large planes. Given the columnar organization of the cortex, this assumption may result in an imprecise estimated of the CSD. Recently, a novel method to estimate CSD has been introduced and it overcomes this difficulty. The method, called iCSD (Pettersen et al., 2006), relies on the explicit inversion of the electrostatic forward solution and assumes the current sources evenly distributed within cylindrical discs of some finite value radius, which enters as parameter in the current estimation.

We applied iCSD to our data and, because we use CSD only for qualitative considerations, we computed the CSD along the in layer plane (1D CSD), assuming that extracellular currents orthogonal to the recording probe have a minimal effect on the spatial sink/source profile. The estimated CSD profiles were qualitatively similar for a choice of the columnar radius ranging between 100 and 200 μm.

To statistically show that CSD is layered, for each experiment we estimated the average CSD strength (absolute value of the CSD) during an oscillation for the cortical layers at a depth of about 650–700 μm and the rest of the layers spanned by the electrodes. The average was over time and space. Then, we compared the experiment-wise samples for the extracted two spatial categories and estimated the Cohen's effect size.

### Energy distribution

The gamma-frequencyband of the signal was selected by pre-filtering the signal with a bandpass of 30–90 Hz, using a constrained least squares finite impulse response filterto avoid non-linear phase distortions (Selesnick et al., 1996). The energy distribution of the recorded signals was estimated by computing a Wigner–Ville distribution, which resolves the distribution of energy over both time and frequency. In particular, we computed an affine-smoothed pseudo–Wigner distribution with a Gaussian smoothing function. This allowed a flexible choice of time and frequency resolutions in an independent manner through the choice of the width of the smoothing functions. After checking the consistency with different choices of the width of the smoothing windows, we chose a window of 512 points for the frequency smoothing, while the time resolution was kept maximal. The Time-Frequency Toolbox software (http://tftb.nongnu.org/) was used for computing the Wigner–Ville distributions.

### Bump fitting

As reported in the Results section, the stimulation-induced transients have the characteristics of damped oscillators. In particular, both amplitude and frequency of the signals are damped. Furthermore, the responses to the shot-like stimuli show a bump-like behavior. To model this time behavior empirically, we chose to fit it by a function proportional to the Gamma probability density function. Our choice was driven, in part, by previous work on fitting bump-like photoreceptor responses (Dodge et al., 1968; Wong and Knight, 1980). The Gamma distribution provides a polynomial increasing term and an exponentially decreasing one—hence the function tends to 0 for *t* = 0 and converges (asymptotically) to 0 for large *t.* In between there is a maximum—for parameter values that place that maximum far from the origin, the function is approximately Gaussian (symmetric), as the maximum moves closer to 0 the bump becomes less symmetric. It is a convenient functional form, and, goodness-of-fit statistics will confirm that it does a good job of removing the slow trend in the multi-electrode response curves.

In formulae, the fitted function is of the form

$$\frac{A}{\Gamma (n)\tau }{\left(\frac{t}{\tau }\right)}^{n-1}e^{-t}/\tau$$

where *t* is the time, Γ is the Gamma distribution (which is only required here as a constant weight—without loss of generality, this term could be subsumed by *A*) and *A*, *n*, *τ* are the three parameters. Specifically, *A* represents the amplitude of the bump, *n* and *τ* are, respectively, the shape and scale parameters of the Gamma distribution corresponding to *A* equal to 1.

Importantly, the *duration* of the bump can be defined and then evaluated by the following formula (Wong and Knight, 1980)

$$\frac{{\left(n!\right)}^{2}2^{2n+1}}{(2n)!}\tau$$

Once *n* and τ have been calibrated. To determine the duration of an oscillation in an experiment for a particular slice, the fitting was performed on the average time course at the recording electrodes on that slice. Moreover, as a further check, we estimated the duration of oscillations with another method, by measuring it as the time interval between the first peak after the stimulation transient and the last peak of the instantaneous power averaged over the recording electrodes. The last peak was determined as the last peak above the noise floor estimated from the average power before stimulation. Both approaches provided concordant results.

### Pair-wise estimator of synchronization

Several methods have been proposed to estimate functional connectivity from multivariate time series (David et al., 2004; Carmeli et al., 2005). They can be subdivided into methods inferring undirected or directed (uni- or bi-directional) functional connectivity (Sato et al., 2006; De Feo and Carmeli, 2008; Sommerlade et al., 2012). Although important, directed functional connectivity is difficult to estimate from real noisy data when the number of time series is high (>10) or the underlying oscillators are not weakly coupled. For these reasons, we opted for estimating undirected functional connectivity, specifically by comparing the phase angles of the interacting oscillatory systems. Indeed, phase coupling is an appealing approach becausephase synchronization phenomena may in general include synchronyof phases but with uncorrelated amplitudes (Pikovsky et al., 2001). In our case, the dominant presence of the gamma rhythm confers meaning to the phase, and makes it natural to use the phase to examine synchronization phenomena. The separation of phase and amplitude was achieved by employing the analytical signal concept, i.e., by means of the Hilbert transform (Rosenblum et al., 2001).

Suppose that we have estimated the phases ϕ^(1)^_*t*_, ϕ^(2)^_*t*_, *t* = 0, …, L − 1, for two signals (1) and (2), respectively. To detect 1:1 phase locking in noisy signals, we can look at the appearance of peaks in the distribution of the difference of the unwrapped phases ϕ^(1, 2)^_*t*_ =ϕ^(1)^_*t*_ −ϕ^(2)^_*t*_,∀*t* (Tass et al., 1998). Hence, an estimator of synchronization (EOS) can be defined (Mormann et al., 2000) as

$${\text{EOS}}^{\left(1,2\right)}={\left|\frac{1}{L}\sum_{t=0}^{L-1}e^{\left(i\phi_{t}^{\left(1,2\right)}\right)}\right|}^{2}$$

EOS^(1,2)^ measures the (time) average phase coherence. By construction, it is zero if the phases are independent one another (ϕ^(1,2)^_*t*_ has a uniform distribution) and is one if the phase difference is constant (perfect phase synchronization). Furthermore, so defined, EOS^(1,2)^ reflects both zero phase lags as well as non-zero phase lag coupling of the phases between two signals.

To measure only phase lagged coupling, one way is to discard phase differences that are centered around 0 mod π. An asymmetry in the distribution of phase differences implies the presence of a consistent, non-zero phase lag between the two signals. An index of the asymmetry of the phase difference distribution is given by Stam et al. (2007)

$${\text{PLI}}^{\left(1,2\right)}=\left|\frac{1}{L}\sum_{t=0}^{L-1}\text{sign}(\phi_{t}^{\left(1,2\right)})\right|$$

when the phase difference ϕ^(1,2)^_*t*_ is bounded in the interval − π < ϕ^(1,2)^_*t*_ ≤ π. The phase lag index (PLI) ranges between 0 and 1. A PLI^(1,2)^ of 0 indicates either no coupling or coupling with a phase difference centered around 0 mod π. A PLI^(1,2)^ of 1 indicates perfect phase locking at a value of ϕ^(1,2)^_*t*_ different from 0 mod π. Recently, the weighted phase lag index (WPLI) has been introduced and shown to possess increased sensitivity compared to the PLI (Vinck et al., 2011). The WPLI is based on the imaginary part of the cross-spectra of the multivariate signals. Considering the difficulty to reliably estimate cross-spectra from very short signals (Böhm and von Sachs, 2009), we opted for the PLI approach only. Furthermore, formulating the WPLI in terms of wavelets would represent an attractive solution, however, such a methodological development is out of the scope of this work.

To investigate the synchronization dynamics during an oscillation, we computed the indices of synchronization (EOS and PLI) on windows of 50 ms shifted of 2 ms. That window of 50 ms allows to include about 3 gamma cycles and corresponds to 1000 samples points. From a statistical estimation point of view that sample size is large enough to reliably estimate the EOS (small variance and bias), which is basically a second order statistic of the distribution of the considered directional variable (or, concentration of the mean direction of the phase difference) (Mardia and Jupp, 2000).

Subsequently, for each pair of signals, to decide whether the stimulation induced synchronization was not explained by noise, we compared the values of synchrony (EOS or PLI) at each time window with the 99% percentile of the values obtained in a pre-stimulation time period of 0.5 s. If the former value exceeded the latter, we declared the synchronization between that pair as existing or significantly non null. To study the robustness of our analysis with respect to the choice of the threshold, we repeated the analyses with the 95% percentile as threshold value. We obtained similar patterns, and so, in the following, we report only the findings obtained with the 99% percentile threshold. Finally, although the distribution of baseline values is key to determine whether the stimulation-induced EOS change is present, it also seems important to take the actual EOS values into account. In this work, we focus only on the presence/absence of synchronization, without investigating the effect size of the EOS changes.

## Results

### Electrically evoked gamma oscillations in cortical slices from young mice

**Figure 1:**
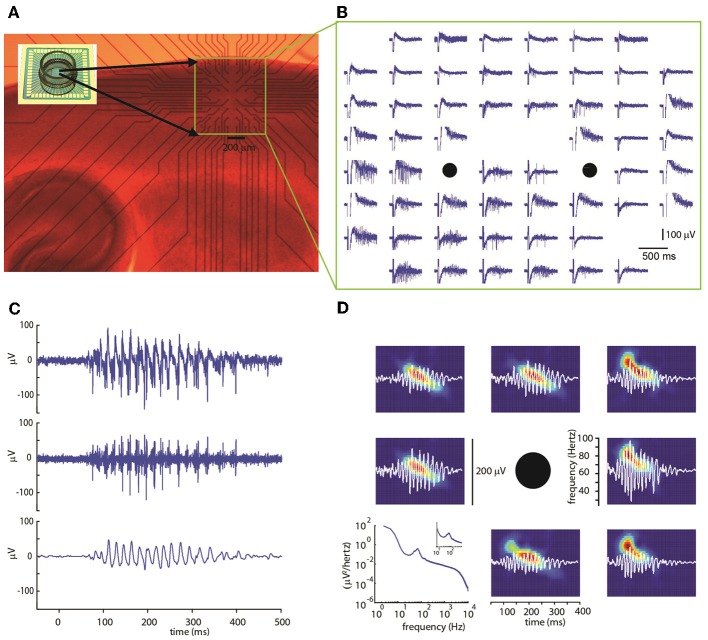
**(A)** Photomicrograph of a sagittal slice of mouse cortex on a multi-electrode array (inset image) with a 60 electrode multi-electrode array. The region containing the electrode is marked by a yellow rectangle. **(B)** Extracellular voltage signals recorded by each electrode. A stimulation consisting of five voltage pulses was simultaneously delivered at the two electrode marked by black circles. Note the evoked gamma oscillation lasting about 400 ms concentrated mostly in the lower electrode rows. **(C)** Extracellular voltage signal for a representative electrode after artifact removal (top, see Methods). High-pass-filter at 100 Hz (center) shows multi-unit activity. Band-pass filter between 30 and 90 Hz (bottom) reveals gamma-frequency oscillations in the local field potential (LFP). **(D)** Representative spectrograms (color coded graphs; each panel is rescaled between its minimal—blue—and maximal value—red) of band-passed signals (white) recorded in the electrodes surrounding the stimulus location (black circle). Typically, bursts start at frequencies around 80 Hz decaying to about 40 Hz within a few hundred milliseconds. In bottom left panel, the power spectrum averaged over all electrodes (*n* = 439), trials (*n* = 67), and slices (*n* = 19) is shown.

Experiments were carried out on 19 sagittal cortical slices from P8-12 mice. The electrical activity in the somatosensory cortical area was evoked and measured using multi-electrode arrays (MEAs) with 60 electrode terminals arranged in an 8 × 8 grid and with an inter-electrode distance of 100 or 200 μm with its top aligned at about 150 μm below the cortical surface. Using this configuration, it was possible to cover most cortical layers (Figure 1A). The spontaneous activity of the network was very low, consisting of only occasional, isolated action potentials (not shown).

**Figure 2:**
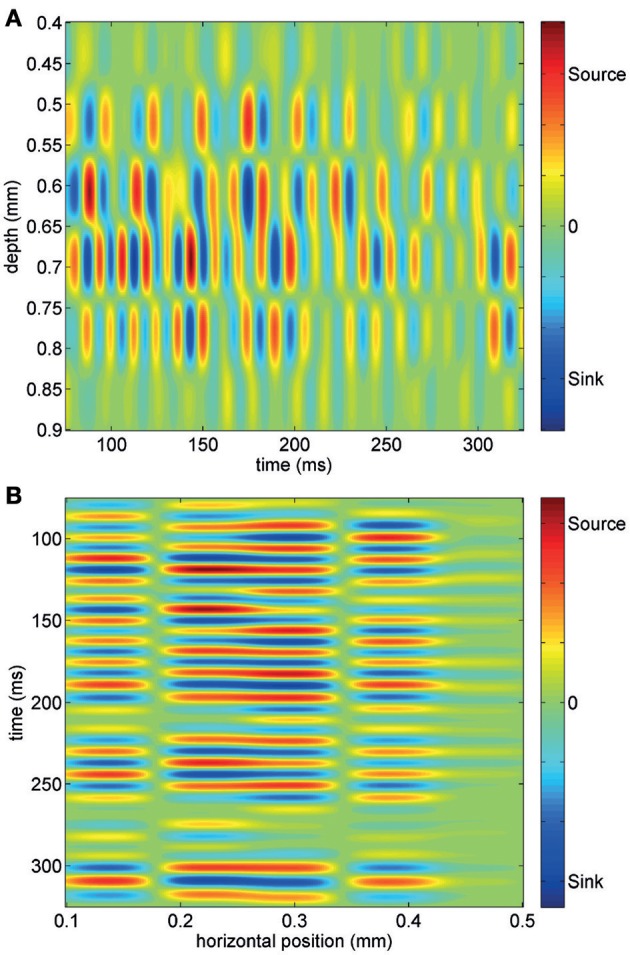
**Current source density analysis of an evoked gamma burst across one column of electrodes (A) and one row of electrodes (B).** Note that most intense sink-source alternation are observed at a depth of about 650–700 μm and that the spatial layering of CSD is observed only across the column.

Using short trains of electrical stimuli (5 bipolar pulses at 200 Hz, see Methods) applied at one or more loci in the network (Figure 1B), we were able to evoke oscillatory network activity in the gamma frequency band (30–90 Hz, Figures 1C,D). Stimulations with different spatial profiles were applied every 30 s and a series of at least 6 trials was collected for each different pattern of stimulation (see Methods). The response of the network to the electrical stimulations was composed of action potentials (multi-unit activity) superimposed on a fluctuating local field potential (LFP, Figure 1C). Only stimuli applied to the central cortical layers (located about 400–600 μm below the cortical surface) were able to evoke gamma oscillations. Oscillations spread to upper and lower layers but were never detected within 200 μm from the cortical surface. The pattern of inward and outward currents was spatially distributed with the most salient sinks and sources located at a depth around 650 μm, as detected by CSD analysis (Figure 2A). Such layering effect was observed only when performing CSD along cortical columns (see Figures 2A–B). The average CSD at the cortical layers of interest was significantly higher than the other layers at *p* < 0.05 with a (large) effect size of about 0.8 (see Section Current Source Density for details).

In contrast, when electrical stimuli were applied in the highest or lowest layers (i.e., less than 200 μm or more than 700 μm from cortical surface) no oscillatory activity was induced and stimulus-evoked activity occurred as isolated APs (not shown). While oscillations could be reliably evoked and measured in slices from P8–12 mice, only occasionally was it possible to measure oscillations at P13–15 (2 out of 15 slices; these data were not included in the analysis).

### Trial-to-trial variability of evoked gamma oscillations

In order to analyze the temporal profile of electrically-evoked gamma oscillations, we used the Hilbert transform to calculate the instantaneous frequencies and amplitudes and the Wigner–Ville energy in the 30–90 Hz band as an index of instantaneous oscillation power (see Methods for details).

**Figure 3:**
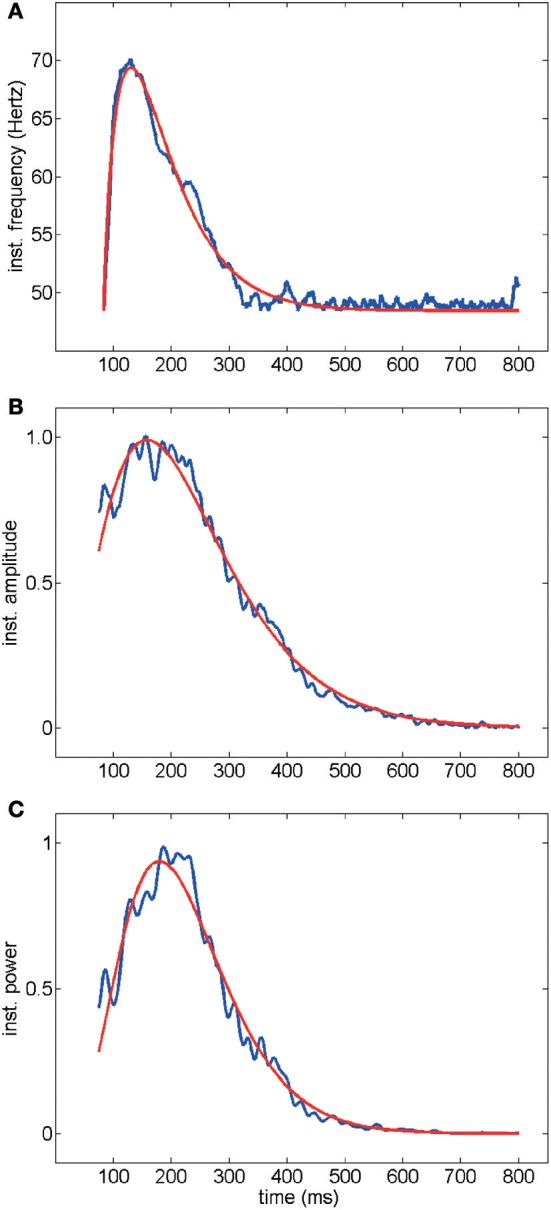
Time course of instantaneous frequency **(A)** normalized amplitude **(B)** and normalized power (Wigner–Ville method, **C**). Average over all electrodes, trials and experiments are shown in blue. Red curves represent fits with gamma distributions (goodness of fit *R*^2^> 0.98). The electrical activity in the first 75 ms following stimulation was not included because of the stimulation artifact transient.

Average time courses of instantaneous frequency, amplitude and Wigner–Ville power are shown in Figure 3 (averaged over the entire data set acquired and analyzed in this work, i.e., 439 electrodes, 67 trials and 19 slices): note the agreement between the fitted gamma distribution “bump” and the data. Typically oscillations started at a frequency of up to 80 Hz decaying down to 40 Hz in a few hundred milliseconds (Figures 3A, 1D). Amplitude and power similarly showed a fast rise followed by a slow decay (Figures 3C,D, 1D). Good fits of the average time courses shown in Figure 3 were obtained with a gamma distribution (see Methods).

**Figure 4:**
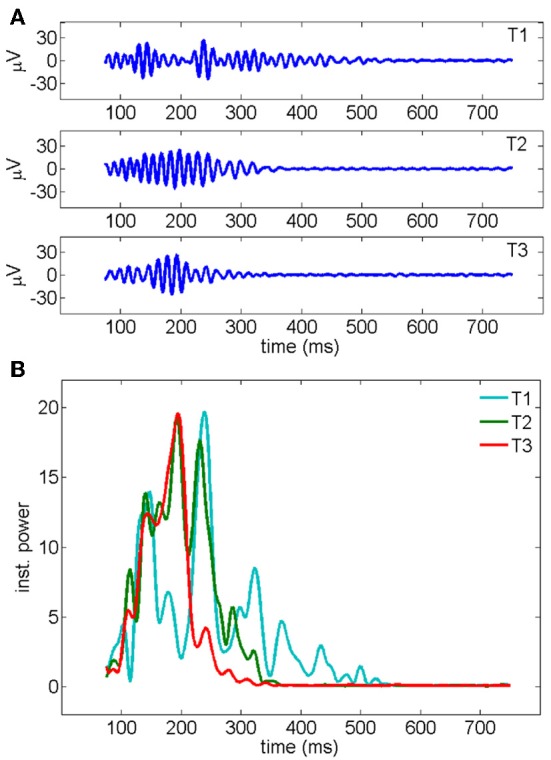
**(A)** Gamma oscillations recorded by a representative electrode across three consecutive trials of stimulation. The signal, measured in μ V, has been band-pass filtered in the 30–90 Hz frequency band (see Methods). **(B)** Instantaneous oscillatory power measured for the three different trials shown in a (y-axis unit: μ V^2^/s). The average over all the 19 electrodes is shown. First, second and third trials are represented, respectively, in cyan, red, and green.

Changes in the temporal dynamics of the oscillations were observed in successive responses to the same stimulus. A representative example of electrical recordings (from one electrode) and the instantaneous power profile (averaged over all 19 electrodes) across consecutive trials is shown in Figure 4.

**Figure 5:**
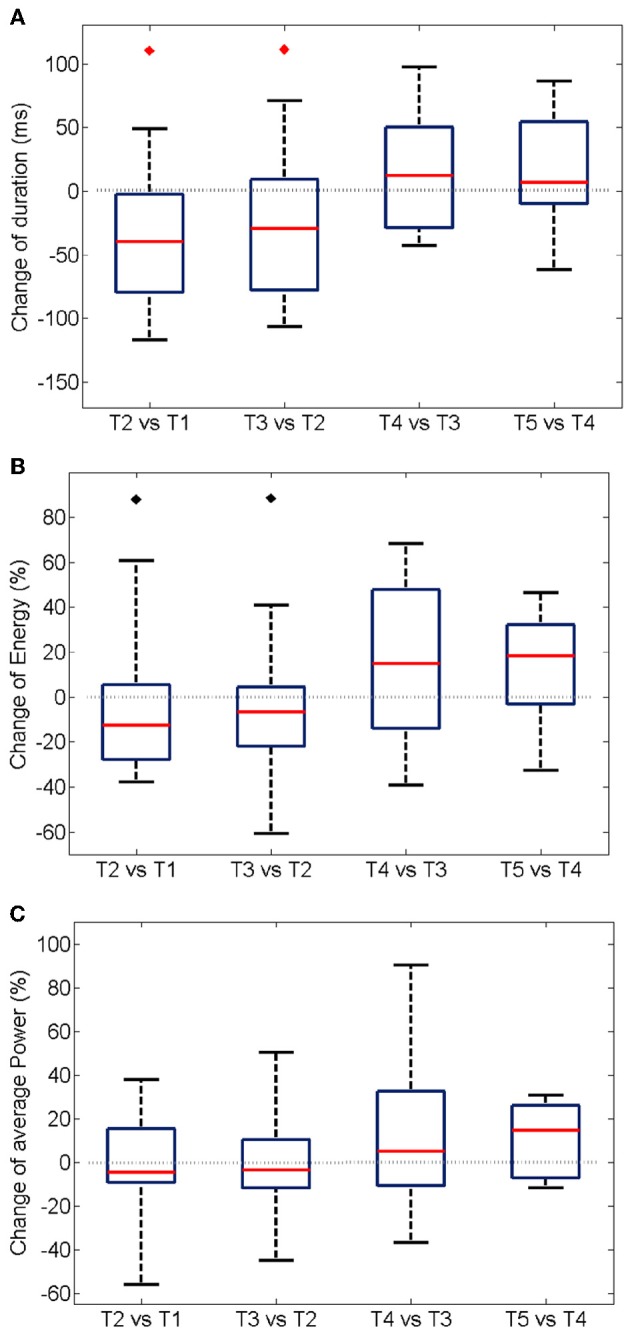
**Trial-wise changes in duration (A), energy (B), and power (C) of the oscillatory responses.** The plots summarize the One-Way Analysis of Variance data from 19 experiments (see Methods). For each experiment, the energy was first computed integrating the instantaneous values over the duration of the oscillation (see Section Energy Distribution for details). An average over all the electrodes was additionally calculated. The boxes have lines at the lower quartile, median, and upper quartile values. The whiskers extend from each of the boxes to show the extent of the rest of the data. The average power at each experiment was modeled as the mean over the recording electrodes of the total energy divided by the duration of the oscillation. The median of the energy is significantly reduced (*p* < 0.05) in the first two inter-trial variations only when considering experiments with a shortening of the oscillation period (noted by black diamonds). The duration of the oscillation period decreases significantly in the second and third trial of stimulation (*p* < 0.05, red diamonds).

The trial-to-trial variation of duration and energy of the oscillations upon the repetition of the same stimulation is summarized in Figure 5 for 19 experiments. A significant shortening of the oscillatory response during the first three trials (Figure 5A) is accompanied by a decrease in the total energy of the oscillation burst (Figure 5B).

Interestingly, no statistically significant change was measured for average power of the oscillations, i.e., the total energy divided by the duration of the gamma burst (Figure 5C), over successive trials suggesting that the *rate* of expenditure of energy during the of gamma bursts is consistent across repeated stimulations. Similarly, no changes in the average frequency and amplitude were observed (not shown).

### Trial-to-trial variability of functional connectivity and spatial synchronization

**Figure 6:**
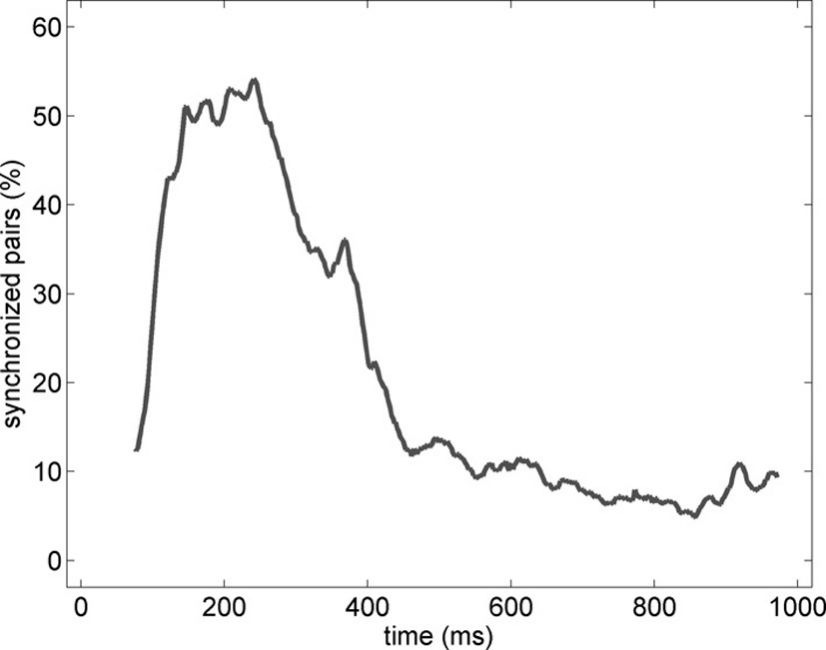
**Percentage of synchronized pairs established during the post-stimulus period for the average of the entire set of experiments (*n* = 19).** All possible connections between the subset of electrodes recording gamma oscillations were considered. We refer here to a synchronized pair if the EOS value of the corresponding pair is higher than the threshold computed from the pre-stimulus time (see Section Pair-Wise Estimator of Synchronization for details).

In order to characterize the spatial properties of the network synchronization, we used a pair-wise approach and a standard bivariate EOS (respectively, EOS and PLI, see Section Pair-Wise Estimator of Synchronization). In addition, the pair-wise approach allowed us to translate the synchronization between two spatial locations in to an active functional connection. Therefore, for each pair of electrodes recording gamma oscillations, the EOS/PLI values during the non-stationary oscillation were used to determine the presence or absence of synchrony/connection. The threshold for determining the functional connection was established as the 99th percentile of the EOS/PLI before the stimulus (see Section Pair-Wise Estimator of Synchronization for details). Significant synchrony was found only with the EOS estimator and the average number of synchronized pairs showed a bump-like time-course (see the example in Figure 6).

**Figure 7:**
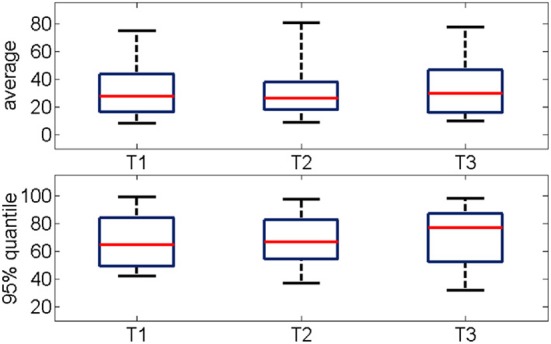
(**Top**) Percentage of functional connections averaged over the all oscillation period for three consecutive trials (*n* = 19). (**Bottom**) Distribution of the 95% quantile of the data shown in the top panel. The networks are mostly not fully connected. The median values range from 30 to 50%, the 95% quantile range from 60 to 90%.

The average percentage of synchronized pairs across the entire duration of the oscillation was about 30% with no significant changes between successive trials (Figure 7 top panel). Given the large variability in the time-course of functional connectivity that was computed on a moving window (see Section Bump Fitting for details), we calculated the 95% quantile of the percentage of pair-wise connections during a gamma burst (Figure 7 bottom panel) observing that it ranged between 60 and 90%. This further estimation confirmed that during most of the duration of the event, the network was not in a fully connected state.

Next, given the anatomical and functional columnar organization of the neocortex, we investigated the spatial patterns of functional connectivity of the gamma oscillations along the columnar (i.e., vertical) direction and the horizontal directions. For all experiments and trials, the average percentage of functional connections within a gamma burst was partitioned according to these two spatial directions and the percentage was obtained by normalizing to the maximum possible number of connections for a given direction.

**Figure 8:**
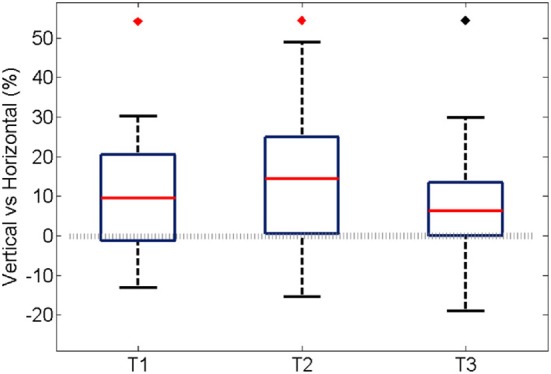
**For all experiments and trials, the average percentage during an oscillation of the pair-wise connections was partitioned according to their spatial direction that is vertical, horizontal, and cetera.** In this case, the percentage was obtained by normalizing to the maximum amount of connection along the corresponding spatial direction. Here we show the difference between the vertical and horizontal ones, for each trial. In the first two trials the median is significantly different from zero (*p* < 0.05, Bonferroni corrected for multiple comparisons, the inherent comparisons here are between the three trials), shown with a red diamond. The median value is about 9 and 14% respectively. The third trial has a median different from zero if the level of significance is not Bonferroni corrected, shown with black diamond. The median value is about 6%.

**Figure 9:**
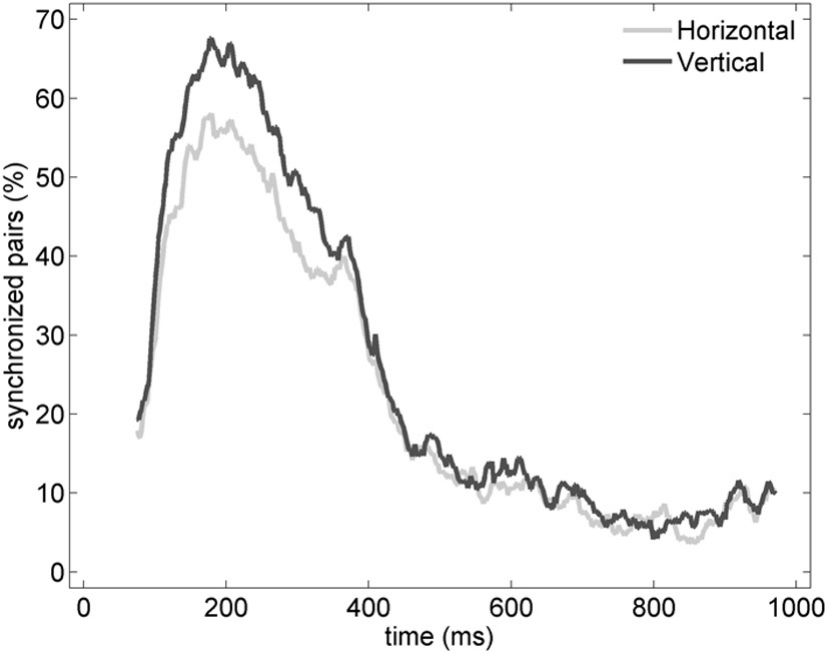
**Percentage of vertical (black) and horizontal (gray) synchronized pairs established during the post-stimulus period for the average of the entire set of experiments (*n* = 19).** All possible vertical and horizontal connections between the subset of electrodes recording gamma oscillations were considered. Similarly to Figure 6, synchronization between pairs was determined by thresholding EOS values based on the pre-stimulus condition (see Section Pair-Wise Estimator of Synchronization for details).

**Figure 10:**
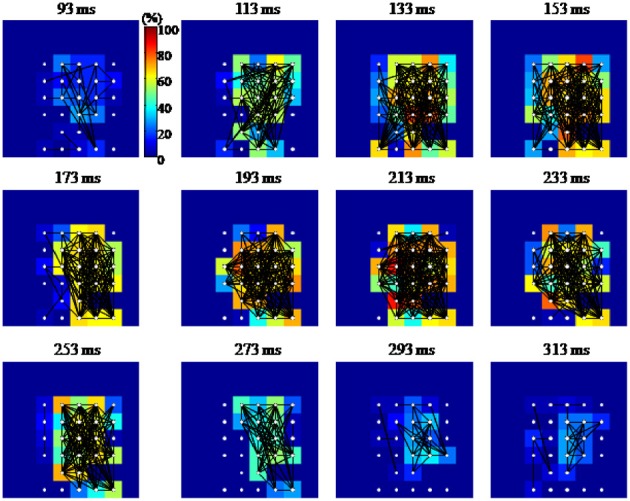
**Graphical representation of the functional connectivity dynamics for an evoked gamma oscillation.** The 8 × 8 pixel images represent the MEA layout (inter-electrode distance 100 μm; top and bottom rows of electrodes were located, respectively, 100 and 800 μm below the cortical surface). Each node or electrode in the network is represented with white filled circles, while a black line is drawn between functionally connected pairs of nodes. Each pixel is color coded according to the percentage of edges impinging to that node (equivalent to the node degree, in graph theoretical jargon). Functional connections are estimated in 50 ms time windows centered at the time highlighted at the top of each image. The shown temporal windows cover the entire duration of the gamma burst. Although densely connected, the network shows a tendency to have denser edges along the columns of the array.

**Figure 11:**
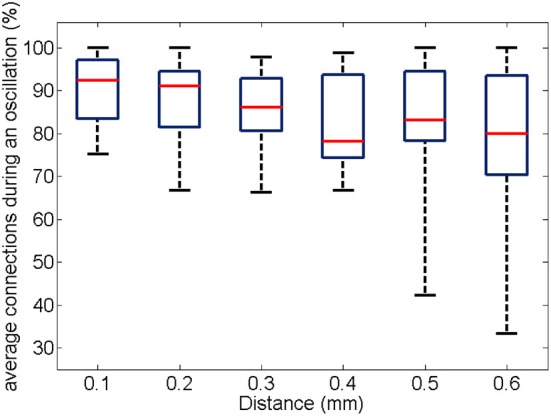
**For all experiments and trials (respectively, 19 and 3), the box plot shows the number of pair-wise connections as a function of distance.** The percentage is obtained by normalization to the number of possible electrode pairs at the given distance.

Similarly to the anatomical and functional organization, the number of functional connections in the columnar direction was higher than the horizontal direction for all trials (Figure 8, level of significance *p* < 0.05, corrected for multiple comparisons along the trials) apart from one case where the *p*-value slightly exceeded the level of significance, with a median value ranging around 6–14%. The functional connectivity anisotropy persisted during the whole gamma burst occurrence and vanished at the end of it (Figures 9, 10). The relation between connectivity and distance is reported in Figure 11. Analysis of variance quantified a significant decrease at 400 μm. We remark that the network is not fully connected although a consistent number of pair-wise connections are still operating at long distances.

**Figure 12:**
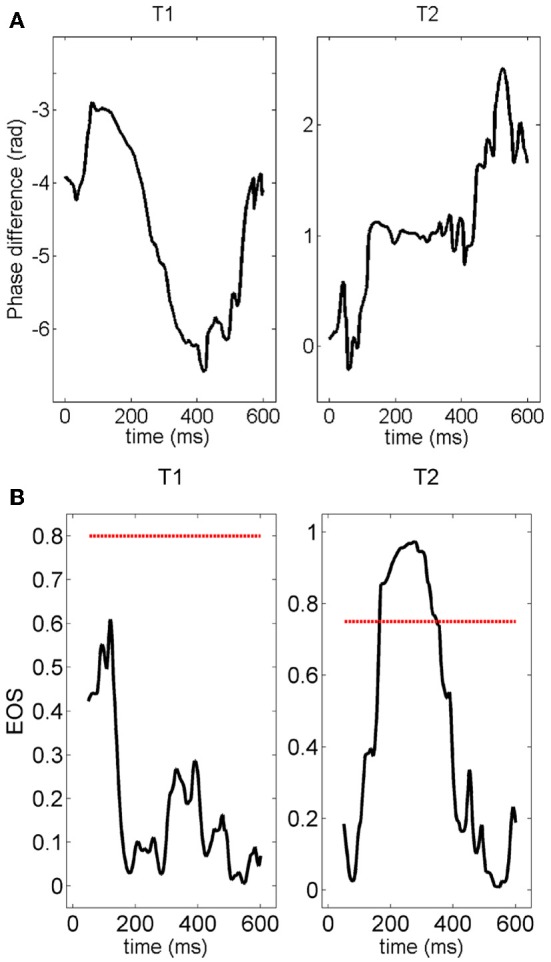
**(A)** Time course of the phase difference (measured with the Hilbert transform) for a given pair of electrodes. The phase difference is normalized by 2π —vertical axes in these plots are multiple of 2π radians. The plateau appearing during the second stimulation (right panel) clearly indicates the presence of synchronization. The value of the phase difference at the plateau is about 2π. **(B)** Time course of the EOS value for the phase differences plotted in **(A)**. The red line represents the threshold computed from the distribution of EOS values during the pre-stimulus time. During trial T1, EOS never goes above the threshold, while EOS is higher than the threshold for several milliseconds during trial T2, demonstrating the appearance of synchronization.

Next, given the temporal and inter-trial variability of the pair-wise connection described above, we quantified the number of connections which persisted, appeared or disappeared across different trials. Figure 12 shows the phase difference of a representative electrode pair at a distance of 400 μm in two different trials. Although in both trials clear gamma oscillations could be recorded in the two electrodes (not shown), synchrony was clearly observed in the second trial (constant phase difference, right panel) but was not present in the first trial.

**Figure 13:**
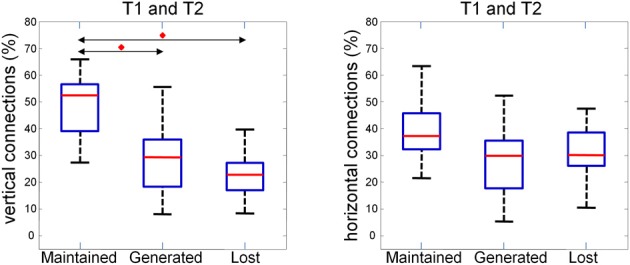
**Average number of maintained, generated and lost connections in two consecutive trials for vertical (left) and horizontal (right) connections.** The percentage is obtained by normalizing to the sum of maintained, generated, and lost connections. We compared the three distributions with a repeated measure Wilcoxon signed rank test (planned comparisons), corrected for multiple comparison (Bonferroni), and found the maintained vertical connections to be significantly (*p* < 0.05, red diamonds) higher than the generated and the lost one.

This observation is quantified and summarized in Figure 13 for vertical (left panel) and horizontal connections (right panel). Given two consecutive trials, about half of vertical connections were maintained while about one quarter of them were generated or lost. In the case of horizontal connections, maintained, generated and lost connections each comprised about one third. Importantly, the maintained vertical connections were significantly higher than the horizontal connections. This result suggests that the spatial pattern of synchrony is shaped upon stimulus repetitions with a more stable columnar than horizontal connectivity.

Based on this observation, we studied the possibility that maintained, generated and lost connections follow a random process. Given the probability *z* and (1 − *z*), respectively, of having and not having a connection in a given trial, *z*^2^ defines the probability to have the same connection in the following trial while *z*(1 − *z*) the probability to loose the connection. The probability of generating a connection will also be *z*(1 − *z*). We tested the null hypothesis that these two probabilities are the same and drawn from a multinomial distribution (random model) using a multinomial test and a Chi-square goodness of fit test. For each experiment the null hypothesis could not be rejected suggesting that a random process underlie the dynamic reconfiguration of connections.

### Excitatory and inhibitory synaptic pathways mediating the occurrence of gamma oscillations

In order to study the excitatory and inhibitory synaptic pathways underlying the gamma oscillations, we tested several drugs (summarized in Table 1). Among them, clearly reproducible and stable results across the age of the animals used (P8–12) could be observed only for carbachol, Gabazine, APV and mefloquine and their effect is discussed in detail below. The effect of the application of the other drugs was too variable to allow a clear quantification, probably due to the fact that the animals used were still in a critical period of development when rapid developmental switches in the mechanisms underlying oscillations can occur (Dupont et al., 2006).

**Figure 14:**
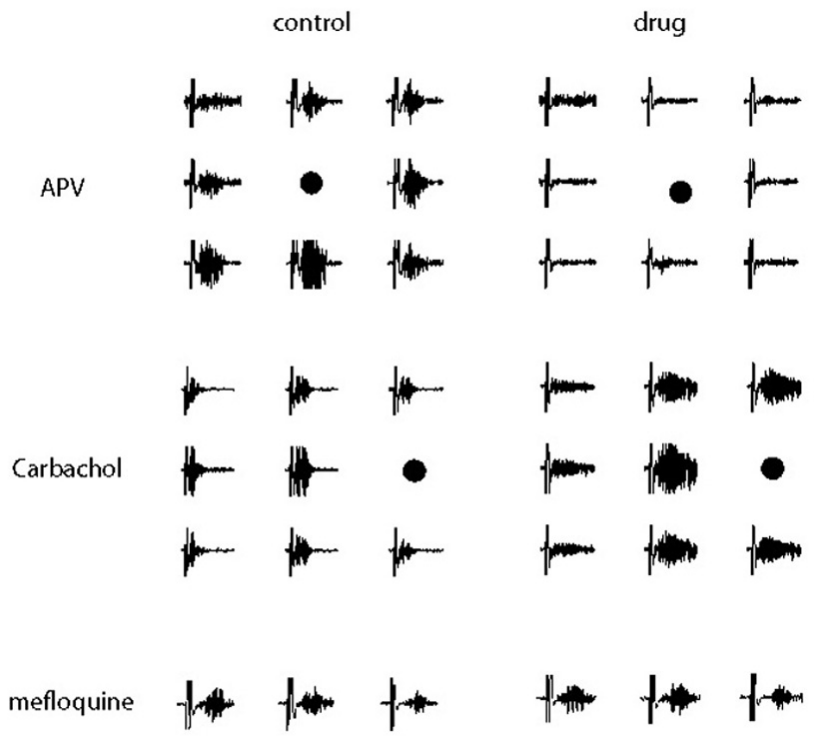
**Electrical recordings of the gamma oscillation evoked in a subsets of electrodes neighboring the location of the stimulation (marked by a black point) in control conditions (left) and during drug perfusion (right).** Different synaptic pathways underlying the occurrence and the dynamics of gamma oscillations were tested, respectively, APV (50 μ M) was used to block glutamatergic excitation mediated by NMDA receptors **(Top)**, carbachol (50 μ M) to activate excitatory pathways meditated by acetylcholine receptors **(Middle)** and mefloquine (25 μ M) to block gap-junction transmission mediated by the connexins 36 and 50 **(Bottom)**. A time window of 600 ms is shown, the vertical axes are between −50 and 50 μ V.

When glutamatergic excitation mediated by NMDA receptors was blocked by bath application of APV (50 μM), gamma oscillations were completely blocked (*n* = 5 slice, Figure 14) and they could be restored after the wash out of the drug.

When excitatory pathways meditated by acetylcholine receptors were activated by bath application of carbachol (50 μM), in 71% of the trials we observed oscillations with longer durations invading a larger portion of the circuitry (Figure 14). In contrast to hippocampal slices where bath application of carbachol induce spontaneous gamma oscillations over a very long time (in the order of dozens of minutes, Fisahn et al., 1998), the effect of carbachol vanished about 20–30 min after the beginning of the drug perfusion, probably due to the desensitization of acetylcholine receptors, similarly to what has been previously reported (Kilb and Luhmann, 2003).

Blockage of the GABAergic transmission mediated by GABA-A receptors by application of Gabazine (10 μM) disrupted gamma oscillations increasing the chance of spontanenous epileptic—like events (not shown).

Since several models hypothesize an important role for electrical synapses in the establishment of the network synchrony occurring during gamma oscillations, we applied mefloquine (100 μM) to block gap-junctions mediated by the connexin36 and 50. However, gamma oscillations were not blocked by mefloquine (Figure 14) but we cannot exclude the possibility that electrical synapses mediated by other connexins or pannexins might not play an important role in establishing the network synchrony underlying the oscillations.

## Discussion

We characterized electrically evoked gamma oscillations in the somatosensory cortical area of juvenile mice (P8–12) in slices. Gamma oscillations occurred in bursts starting at high frequencies (~80 Hz) and gradually moving toward lower frequencies (~40 Hz) in few hundreds milliseconds. Similarly to what previously reported in rodents at the end of the first postnatal week, gamma oscillations required GABAergic transmission mediated by GABA-A receptors (Minlebaev et al., 2011) and glutamatergic excitation mediated by NMDA receptors (Dupont et al., 2006). In addition, we observed that gamma oscillations power was enhanced by the activation of excitatory pathways meditated by acetylcholine receptors, in agreement to what previously reported (Buhl et al., 1998; Dupont et al., 2006). In contrast to hippocampal circuits (Buhl et al., 2003), gamma oscillations were not abolished or reduced in power when gap-junction transmission mediated by the connexin 36 and 50 was blocked.

In our study we observe that gamma oscillations can change both in terms of duration and total energy but maintain a constant global power. Shortening is unlikely to be due to long-term changes in stimulus electrodes. This observation is repeatable, and happens well after the shocks.

In our study, instantaneous synchronization between different regions reveals functional connections that vary within the same oscillatory event and from one repetition to another. The spatial organization of the functional connectivity reflects the columnar organization of the cortex with more vertical connections than horizontal. About 50% of the vertical connections are kept from trial to trial while 25% are lost and generated with repetition of the same stimulus. About 30% of the horizontal are kept from trial to trial while 30% are lost and generated with repetition of the same stimulus. This result shows that part of the functional connectivity is reshaped from trial to trial in an itinerant fashion supporting the idea that the variability of the neuronal responses allows the network to explore different states. This might represent a mechanism of learning and a similar phenomenon has been described in dissociated cultures of cortical neurons (Shahaf and Marom, 2001). Variability can be determined by spontaneous fluctuations and can represent a mechanism of binding different region depending on the underlying network state that can be determined by other cortical region rather than spontaneous activity *in vivo* physiological conditions (Fries, 2005, 2009).

The electrically evoked gamma transients presented in this study are very similar to what described by Minlebaev et al. (2011) *in vivo* in the neonatal rat cortex where the deflection of a whisker triggered a gamma response of few hundreds milliseconds in the corresponding barrel in the first postnatal week. In that study, from the second postnatal week the maturation of inhibition within the cortical circuit reduced the entraining influence of the thalamic input in shaping oscillatory events in favor of the local inhibitory circuits. We hypothesize that the synchronous excitation of the deeper layers of the cortical circuit induced by the electrical stimulation can mimic the natural excitatory thalamic input (which is similarly delivered to the deeper cortical layers). The rhythm of the gamma oscillation is locally imposed by the inhibitory circuit. In addition, the low electrical activity and the lack of oscillations in the upper cortical circuits which are the less developed at that stage of circuit maturation, result in a less effective horizontal communication and synchronization of the cortical circuits.

The distinction between vertical and horizontal functional connectivity (Figures 8–10) is consistent with anatomy and indicates that the main driving cause of the observed connectivity is not bulk diffusion. This is additionally supported by the trial-to-trial variability of functional connectivity (Figures 11, 12) since functional connections can be present or absent in different trials of the same stimulation. Nonetheless, we are cautious about interpreting the exact structures of functional connectivity, which we observe. Although graph-theoretic metrics (such as mean shortest path-length) are expected to change with changes in synchrony (e.g., mean shortest path length decreasing as synchrony), it does not seem useful at this stage to interpret variations amongst trials in terms of particular network structures (Bialonski et al., 2010), especially because of the variable number of nodes in the networks (between 19 and 32) which might bias that kind of metrics. The highly variable and apparently random nature of fluctuations in multi-electrode evoked gamma oscillations is consistent with a very complex system. The challenge for the future will be to reconstruct such behavior in accurate biophysically-based models and understand its implications for the information processing capabilities of gamma oscillations in the cortex (Woolrich and Stephan, 2013). A key methodological development will be approaches to determine in a reliable way the number and location of the bioelectrical sources prior to the inference of the model parameters. We expect that one of those approaches will be to derive robust priors for the parameters of the biophysical models from exploratory functional connectivity analysis as the one described in this work.

### Conflict of interest statement

The authors declare that the research was conducted in the absence of any commercial or financial relationships that could be construed as a potential conflict of interest.
